# Turmeric-black pepper-honey nanoemulsion formulation and antiulcerogenic effect evaluation against ethanol-induced gastric ulcers in rats

**DOI:** 10.1371/journal.pone.0317899

**Published:** 2025-01-22

**Authors:** Amirah Adlia, Cynthiana Christabella Aslan, Lia Safitri, I. Ketut Adnyana

**Affiliations:** School of Pharmacy, Bandung Institute of Technology, Bandung, Indonesia; National Institute of Food Technology Entrepreneurship and Management, INDIA

## Abstract

Gastric ulcer is a common disorder of the digestive system. The combination of turmeric and honey is known to treat stomach ulcers. However, curcumin, an active component in turmeric, has limitations, i.e., poor water solubility and low oral bioavailability. Therefore, turmeric and honey were formulated into a nanoemulsion with black pepper to enhance curcumin bioavailability. The study followed a systematic approach to optimize the nanoemulsion formula, determine stability, and evaluate ulcer healing activity in rats with ethanol-induced gastric ulcers. Nanoemulsion was prepared using a low-energy emulsification method called emulsion phase inversion (EPI). Two stability evaluations were carried out, i.e., storage and freeze-thaw stability tests. The organoleptic, droplet size, polydispersity index, pH, viscosity, and curcumin content of the nanoemulsion were evaluated. Male Wistar albino rats were induced with 96% ethanol for six days. The rats were divided into six groups, i.e., healthy control, ulcerated control, omeprazole, two different doses of turmeric, honey, and black pepper nanoemulsion (NTBH1 and NTBH2), and turmeric and honey nanoemulsion (NTH). The antiulcer activity was determined by measuring the ulcer area, ulcer index, curative index, ulcer severity score, and histology. The best formula with the smallest droplet size, i.e., 144.6±3.8 nm, was obtained from the nanoemulsion using Tween 80 as surfactant, glycerin as cosolvent, and sodium alginate as viscosity enhancer. The result showed that the nanoemulsion was stable after being stored at 25 and 40°C for four weeks and after six cycles of freeze-thaw test. The ulcer index of the ulcerated rats from the lowest to the highest, i.e., NTBH2, omeprazole, NTH, and NTBH1. In conclusion, the nanoemulsion developed in this study containing turmeric, honey, and black pepper holds promising potential in treating gastric ulcers, offering a hopeful outlook for future treatments.

## Introduction

Gastric is one of the vital organs in the gastrointestinal system, which supports the digestion process mechanically and chemically through the presence of gastric acid and pepsin. In addition, gastric acts as temporary food storage, and gastric acid is a non-immunological defense mechanism against pathogenic microorganisms. When a gastric problem occurs, the digestive process is also disrupted. Consequently, the immune system will be negatively affected due to a lack of absorbed nutrition. One of the frequent problems in gastric is gastric ulcer [1, 2]. A gastric ulcer, which occurs inside the stomach, is a type of peptic ulcer, while peptic ulcers can also occur in the upper portion of the small intestine [2]. In this study, we are focusing on the treatment of gastric ulcers.

Gastric ulcer disease has become a significant health issue with an unchanged mortality rate in the last 50 years [1]. Although the prevalence of *H*. *pylori* has been falling globally over the last 20 years, there has been an increased prevalence of risk factors, including the increased use of nonsteroidal anti-inflammatory drugs (NSAIDs), anti-platelet, and anticoagulant therapy over time, alcohol consumption, and stress [1–3]. It is important to note that increasing age contributes to the increased risk of peptic ulcer bleeding. People over the age of 60 have a 10-fold higher risk of peptic ulcer complications compared to younger age groups [1].

The damaged mucous, submucous, and even muscular layers are observed conditions in gastric ulcers. The pathophysiology of gastric ulcer develops when there is an imbalance between the aggressive factors, causing the over-secretion of gastric acid, and defensive factors, which protect the mucous layer [1, 2]. Gastric acid, pepsin, *Helicobacter pylori* (*H*. *pylori*), NSAIDs, alcohol, and cigarettes are categorized as aggressive factors [1]. Meanwhile, the defensive factors include mucous, bicarbonate, surface epithelial cells, blood flow, prostaglandin, phospholipid, and intracellular pH regulation [1].

Eradicating *H*. *pylori* is more effective than anti-secretory therapy for peptic ulcer patients with *H*. *pylori*-positive [1]. While anti-secretory therapy, including proton pump inhibitors (PPIs) and H2-receptor antagonists, are effective in managing symptoms, their long-term use raises safety concerns. PPIs have been associated with enteric infections [4], cardiovascular events [5], and chronic kidney disease [6], which outweighs the benefits of treating peptic ulcers. Meanwhile, long-term use of H2-receptor antagonists have been associated with reduced absorption of vitamin B12 from food [7].

Currently, there is a growing trend toward using natural products, such as medicinal plants, as alternative or complementary treatments, particularly for long-term therapies. It is believed that long-term use of medicinal plants has minimal side effects compared to conventional drugs. Studies have shown several possible mechanisms of the antiulcer effect of medicinal plants, i.e., antioxidant activity, stimulation of mucosal proliferation, inhibition of acid production and secretion, increased mucus production, and reduction or inhibition of inflammation [8–11]. Nevertheless, most of the potential substances for anti-ulcer were claimed to have gastroprotective effects [8, 10, 11]. The anti-ulcer activity of most medicinal plants was evaluated on in vivo models of gastric ulcers, in which the treatment was administered before the induction of the noxious substances, including ethanol [11]. The ethanol-induced gastric ulcer model is a widely accepted experimental approach for assessing the gastroprotective and ulcer-healing properties of therapeutic agents. Ethanol disrupts the gastric mucosal barrier by increasing oxidative stress, decreasing bicarbonate secretion, and inducing inflammation [12, 13]. These features closely resemble the pathophysiological mechanisms observed in human ulcers, making this model particularly relevant for evaluating therapeutic efficacy [14].

The current clinical treatment for gastric ulcers has a high rate of recurrence and a low rate of cure [15]. Thus, evaluating the efficacy of potential anti-ulcer substances in a pre-established gastric ulcer model is also necessary. The ethanol administration for five days has been observed to produce a histological profile indicative of chronic gastric injury, along with noticeable changes in the plasma membranes of the gastric mucosa [16, 17]. So, this animal model can be used to evaluate anti-ulcer activity in a pre-established gastric ulcer.

A previous study from our group showed the potential effect of turmeric (*Curcuma longa* Linn.) in treating gastric ulcers [9]. Turmeric contains curcumin as the main bioactive compound with several known activities, including antioxidant, anti-inflammatory, chemopreventive, and chemotherapeutic [18, 19]. A better ulcer healing effect was observed when turmeric was combined with honey, and the best effect was observed in the treatment with honey only. Nonetheless, the results also showed that using honey alone decreased the pH of gastric juice, which in the long term can exacerbate the condition and delay healing [9]. This is likely because honey has a low pH [20]. We postulated that the hindered effect of turmeric in the previous study was due to the low bioavailability of curcumin. Therefore, we formulated turmeric with black pepper and honey into nanoemulsion in this study. Black pepper (*Piper nigrum* L.) was used to enhance the bioavailability of curcumin. Piperine, a main compound of *Piper nigrum* L., enhanced the serum concentration, extent of absorption, and bioavailability of curcumin in rats and humans [21]. In another study, black pepper was shown to have a gastroprotective effect [22]. Black pepper has been used in traditional Chinese medicine for treating stomachache, earache, muscular ache, etc. [23].

While extensive research has explored the gastroprotective effects of turmeric, honey, and black pepper individually, limited studies have investigated their combined effects, particularly in nanoemulsion formulation. Moreover, most studies on natural products focus on prophylactic effects rather than therapeutic interventions in pre-established ulcer models [8, 10, 11, 24]. This study aimed to bridge these gaps by evaluating the curative potential of turmeric-black pepper-honey nanoemulsion using a chronic ethanol-induced ulcer model, which more closely mimics clinical scenarios.

Nanoemulsions have emerged as a pivotal drug delivery system due to their ability to improve the solubility, stability, and bioavailability of poorly water-soluble compounds, including curcumin [25–29]. Moreover, several studies have shown that potential active ingredients have enhanced anti-ulcer activity when formulated into nanoemulsion [30–32]. The improved effect of nanoemulsion formula may result from enhanced bioavailability through increased solubility. In addition, the small droplet size provides a larger surface area for interaction with the gastric lining, facilitating more efficient drug absorption [31]. Recent studies have shown that nanoemulsions significantly enhance the pharmacokinetics of curcumin, allowing for better systemic absorption and therapeutic effects in various models of inflammation and chronic diseases [29, 33–35]. A low-energy method was chosen to produce nanoemulsion in this study because this method does not require sophisticated instruments, such as high-pressure homogenizers, colloid mills, sonicators, or microfluidizers [36, 37]. The low-energy method requires a relatively high amount of surfactant, but only simple and slow stirring is used to produce nanoemulsion [38].

Several methods have been developed to produce nanoemulsion using a low-energy approach, such as spontaneous emulsification and phase inversion methods [39]. Nanoemulsion produced using the phase inversion method relies on inducing phase inversion from a W/O to an O/W system [40]. Phase inversion temperature, phase inversion composition, and emulsion phase inversion are examples of this method. In this study, the emulsion phase inversion (EPI) method, based on catastrophic phase inversion, was chosen. Water was titrated into a mixture of oil and hydrophilic surfactant to induce catastrophic phase inversion. EPI was selected because it has been shown in some studies that the EPI method can produce a stable nanoemulsion system [36, 39–41]. The nanoemulsion obtained in this study was further evaluated for its physical stability and anti-ulcer activity in an ethanol-induced gastric ulcer animal model.

## Materials & methods

### Materials

Turmeric and black pepper extracts were provided by PT Sari Alam Sukabumi, Indonesia. Indonesian Randu honey was purchased from PT Mavca Natways Indonesia, Indonesia (BPOM RI MD252110009688). Fractionated coconut oil (caprylic/capric triglyceride 60/40) was purchased from CV Mikaya Makmur Sejahtera, Indonesia. Virgin coconut oil was purchased from CV Al-Ghuroba, Indonesia (BPOM RI MD071119001800288). Tween 80, glycerin, sodium benzoate, sodium alginate, and sodium carboxymethylcellulose (CMC-Na) were purchased from PT Brataco, Indonesia. The curcumin reference standard was provided by MarkHerb, Indonesia.

### Determination of the suitable edible oil to dissolve curcumin from turmeric extract

There are two different types of oil used in this study, i.e., virgin coconut oil (VCO) and fractionated coconut oil (FCO). The maximum solubility of curcumin in each oil was determined using the spectrophotometry adapted from Ahmed et al. with slight modifications [42]. 250 mg of turmeric extract was dissolved in 10 mL of oil. The mixture was stirred and heated at 60°C for 10 minutes, then sonicated at 59 Hz for 20 minutes. The mixture was further centrifuged at 1750 rpm for 10 minutes, and the absorbance of the isolated supernatant was measured at 435 nm using a UV-Vis spectrophotometer (Beckman DU7500i).

### Nanoemulsion formulation

Nanoemulsions were prepared using a low-energy method adapted from Ostertag et al. with slight modifications [36]. The ratio of curcumin and piperine in the formula was set to 33:1. The amount of turmeric and black pepper extracts used in the formulation was determined based on the content of curcumin and piperine in each extract, respectively. To prepare 20 mL of the formula, the turmeric (containing 50 mg curcumin) and black pepper (containing 1.5 mg piperine) extracts were added to 0.4 mL of FCO. Then, Tween 80 and glycerin were added according to the designated formula (Table 1). The mixture was then stirred at 400 rpm for 30 minutes. Furthermore, the aquadest was titrated into the mixture with a flow rate of ±3mL/min while stirring at 500 rpm for 30min. The honey (20% w/v) was subsequently added with dissolved sodium benzoate and sodium alginate or CMC-Na (Table 1). The mixture was then stirred at 500 rpm for 30 minutes. The obtained nanoemulsion was further characterized.

**Table 1:** Nanoemulsion turmeric-black pepper-honey formula optimization.

Formula	Amount in formula (% w/v)			
	Tween 80	Glycerin	Sodium Alginate	CMC-Na
F1G1A	2	4	1.5	
F2G1A	3	4	1.5	
F3G1A	4	4	1.5	
F3G2A	4	8	1.5	
F3G2C	4	8		0.4

### Nanoemulsion characterization

#### Mean droplet diameter and polydispersity index (PDI)

Droplet size and PDI were measured using Delsa™ nano C Particle Analyzer. This instrument can measure particle size from 0.6 nm to 7 μm with the optimum concentration of 0.001% to 40%. Samples were diluted in ultrapure water (1:100) prior to analysis to avoid multiple scattering effects [43].

#### pH

The nanoemulsion pH was determined using a pH meter (Mettler Toledo) at room temperature (25 ± 2°C).

#### Viscosity

The viscosity of the nanoemulsion was measured using a Brookfield viscometer at room temperature (25 ± 2°C), spindle number 2, with a speed of 2.5 rpm [44].

#### Curcumin loading efficiency

The curcumin content in the nanoemulsion was determined by using a UV-VIS Spectrophotometer. First, the nanoemulsion was diluted with methanol and sonicated at 59 Hz for 5 minutes [45]. Subsequently, the mixture was centrifuged at 3500 rpm for 1 minute. The absorbance of the isolated supernatant was measured at 425 nm. The calibration curve was generated from the curcumin reference standard.

### Stability test

#### Storage and accelerated stability test

The storage and accelerated stability tests were conducted for four weeks by storing the nanoemulsion at 25±2°C and 40±2°, respectively [46]. After being stored in both conditions, the nanoemulsion was characterized. The characterization included physical appearance, droplet size, polydispersity index, pH, viscosity, and curcumin content at weeks 0, 2, and 4.

#### Freeze-thaw test

In the freeze-thaw test, the nanoemulsion was stored in the freezer (-20±2°C) and at room temperature (25 ± 2°C) for six cycles [47]. One cycle consisted of 48 hours of storage in the freezer and 48 hours at room temperature. The physical appearance, droplet size, polydispersity index, pH, viscosity, and curcumin loading efficiency in the nanoemulsion were evaluated before the first cycle and at the end of the sixth cycle.

### Animal experiment

#### Evaluation of healing activity in ethanol-induced gastric ulcer in rats

The experiments were performed on adult male Wistar rats (150–200 g). The animals were kept in cages with wood shaving at room temperature with 12h dark/light cycles, fed with standard pellets, and allowed free access to water. Ongoing monitoring and detailed recordkeeping of animal body weight and behavior were done to ensure their welfare throughout the experiment. The animal experiments were approved by the Animal Research Ethics Committee of Institut Teknologi Bandung, Indonesia (Ethical approval No. 02/KEPHP-ITB/03-2021). The animal experiment method was adapted from Hernández-Muñoz et al. with slight modifications [16]. In this study, the animal experiment was done in six days. The gastric ulcer was induced by oral administration of 96% ethanol (5 mL/kg) on days 1, 5, and 6. In addition, the 96% ethanol was administered on days 2, 3, and 4 with a lower dose of 96% ethanol, i.e., 2.5 mL/kg.

To evaluate the gastric ulcer healing activity, 30 animals were randomly divided into six groups, each consisting of five animals. The groups were as follows: healthy control; ulcerated control; positive control (ulcerated rats treated with Omeprazole suspension 20 mg/kg); turmeric-black pepper-honey nanoemulsion low dose (NTBH1; ulcerated rats treated with 1 mL of nanoemulsion/kg of rat’s bodyweight); turmeric-black pepper-honey nanoemulsion high dose (NTBH2; ulcerated rats treated with 2 mL of nanoemulsion/kg of rat’s bodyweight); nanoemulsion without black pepper (NTH; ulcerated rats treated with 2 mL of nanoemulsion/kg of rat’s bodyweight). Omeprazole and nanoemulsion were administered orally daily from day 1 to day 5, together with ulcer induction. Meanwhile, the healthy and ulcerated control groups received only water.

The animals were fasted 12 hours before the first (day 1) and the last induction (day 6). On day 6, 96% ethanol was administered 1 hour before sacrifice. The animals were euthanized by using the carbon dioxide (CO_2_) asphyxiation method [48]. Animals were placed in a chamber filled with 100% CO_2_ at a displacement rate of 10–30% of the chamber volume per minute for 2–3 minutes [48]. The animals were removed from the chamber after observing signs of lack of breathing and faded eye color.

#### Tissue sampling and collection of gastric juice

Surgery was conducted on the mid-sagittal part of the euthanized rats to collect the gastric [9]. The gastric was spread on the surgery board for macroscopic observation. Simultaneously, gastric juices were collected to measure pH. Gastric pH was determined semi-quantitatively using indicator pH [9]. For histological analysis, the gastric tissue was fixed in 10% buffered formalin for 12h, dehydrated, and embedded in paraffin wax.

#### Estimation of gastric ulcer index

The area of gastric and ulcer was measured using ImageJ, so the ulcer and curative index can be calculated [10].


Ulcerindex(UI)=(ulcerarea/gastricarea)x100

$$Curative\;index=(UI_{disease}‐UI_{treatment})/UI_{disease}\;x\;100$$

In addition, scoring evaluation was carried out according to Table 2 [24].

**Table 2:** Gastric ulcer scoring evaluation.

Characteristic	Score
Without lesion (normal gastric)	0
Hyperemia	0.5
Hemorrhagic spots	1
1–5 small ulcers	2
Few small ulcers	3
1–5 small and 1–3 large ulcers	4
Few small and large ulcers	5
Gastric full of ulcers and perforation	6

#### Hematoxylin and eosin staining

Paraffin-embedded tissue samples were sectioned at a thickness of 4 μm, mounted on glass slides, and stained with hematoxylin-eosin [49]. The sections were deparaffinized in xylene and rehydrated through a graded series of ethanol (100%, 96%, 70%) to distilled water. For hematoxylin staining, slides were immersed in Mayer’s hematoxylin solution for 5 minutes, followed by rinsing under tap water for 5 minutes to allow for bluing. The slides were then submerged in eosin solution for 2 minutes. After eosin staining, slides were washed in distilled water, dehydrated through a graded ethanol series (70%, 96%, 100%), and cleared in xylene. Coverslips were mounted with DPX mounting medium. Stained slides were examined under a light microscope to assess tissue morphology and histopathological changes.

### Statistical analysis

The obtained data from this study was presented as average±standard deviation. Statistical analysis was performed using GraphPad Prism 9, employing one-way ANOVA followed by a post hoc test with Tukey HSD. The difference is significant when p<0.05.

## Results

The curcumin solubility was 1.4±0.1 times higher in FCO compared to VCO. Thus, this study used FCO as carrier oil for the nanoemulsion formula. Formula optimization was done by varying the surfactant, cosolvent, and gelling agent concentration. The droplet size was determined to choose the best nanoemulsion formula. The best formula with the lowest droplet size was obtained from F3G2A, with a surfactant-to-oil ratio of 2:1 (Table 3). The obtained turmeric-black pepper-honey nanoemulsion was transparent, with a droplet size of 144.6±3.8 nm (Table 3). Cosolvent also plays an essential role in reducing the droplet size of nanoemulsion. The glycerin concentration in formula F3G2A was twice the concentration of glycerin in formula F3G1A, and the smallest droplet size was found in higher concentrations of glycerin. In addition, two types of gelling agents were used in this study, i.e., sodium alginate and CMC-Na. The smallest droplet size was obtained from the formula using sodium alginate as a gelling agent. Thus, formula F3G2A was pursued as turmeric-black pepper-honey nanoemulsion in this study for further stability tests and in vivo antiulcerogenic effect evaluation.

**Table 3:** The droplet size of optimized nanoemulsion formula.

Formula	Droplet size (nm)
F1G1A	>800
F2G1A	>800
F3G1A	341.1±20.3
F3G2A	144.6±3.8
F3G2C	358.8±12.2

**Fig 1:**
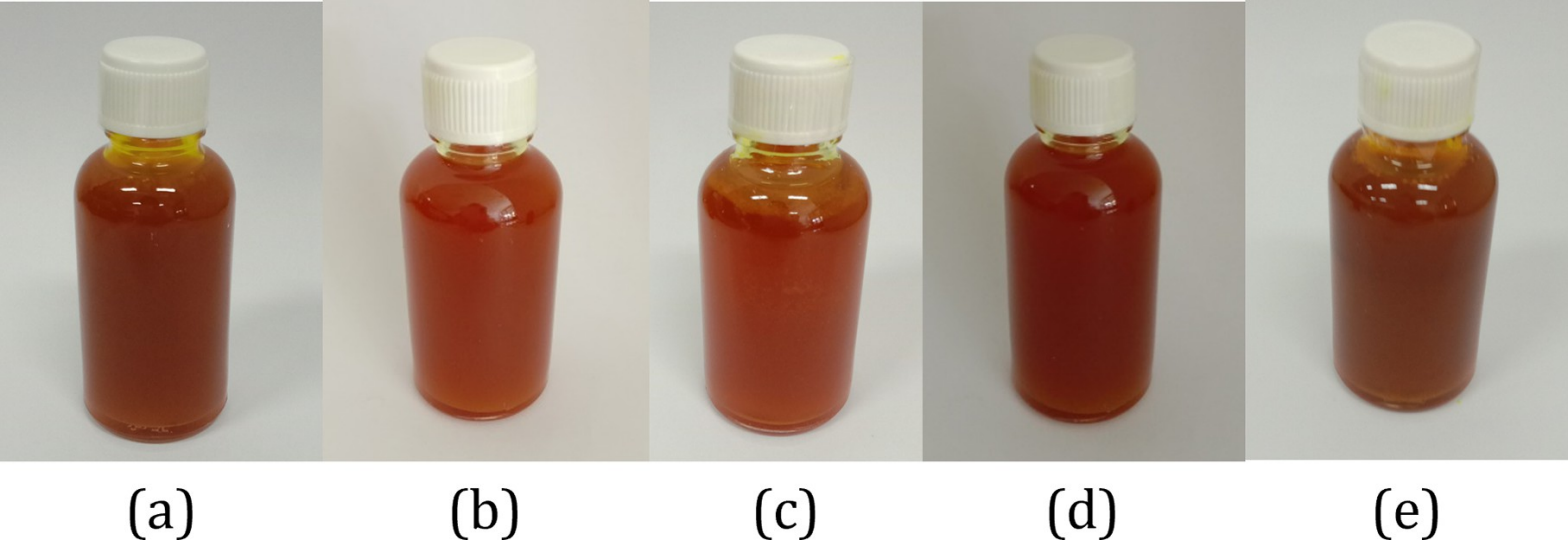
The visual appearance of nanoemulsion in storage stability test. (a) Nanoemulsion appearance on week 0, and (b) after stored at 25°C on week 2; (c) on week 4, and (d) at 40°C on week 2.

The stability tests were carried out using the storage stability test and the freeze-thaw method. The storage stability test was done at 25±2°C and 40±2°C. During the storage stability test, a four-week observation at both temperatures showed no organoleptic changes in the nanoemulsion (Fig 1).

The pH was slightly decreased during 4-week of storage at both temperatures, but the difference was not statistically significant compared to the initial pH (Table 4). The pH fulfilled the requirement of curcumin pH stability, i.e., less than 7, and the preservative used in the formula, sodium benzoate, is active at the pH of 5 [50].

**Table 4:** pH, droplet size, polydispersity index, viscosity, and curcumin content after storage at 25°C and 40°C.

week	pH		Droplet size (nm)		Polydispersity index		Viscosity (cP)		Curcumin (%)	
	25°C	40°C	25°C	40°C	25°C	40°C	25°C	40°C	25°C	40°C
0	5.5±0.1		144.6±3.8		0.30±0.03		744.2±30.3		104.5±1.1	
2	5.3±0.0	5.4±0.0	156.6±6.4^a^	196.2±1.1^a^	0.29±0.06	0.30±0.06	880.1±62.5	395.2±32.4^a^	104.3±3.1	101.1±7.7
4	5.2±0.1	5.4±0.1	168.7±4.2^a^	229.8± 6.2^a^	0.31±0.08	0.29±0.04	850.5±57.3	290.6±53.1^a^	103.8±1.9	99.5±3.4

a = significantly different compared to week 0 (p<0.05)

The droplet size increased significantly at both storage temperatures after 2 and 4 weeks (Table 4). A higher increase was observed at a storage temperature of 40°C. Nevertheless, the droplet size was still in the size range for nanoemulsion, i.e., less than 200 nm [51]. The polydispersity index, however, did not change significantly after four weeks of storage at both temperatures. The nanoemulsion system can also maintain the stability of curcumin at both storage temperatures (Table 4). However, the viscosity of the nanoemulsion decreased significantly at 40°C after two weeks and four weeks of storage (Table 4).

**Fig 2:**
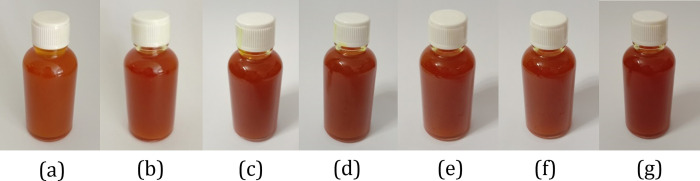
The visual appearance of nanoemulsion in the freeze-thaw test. The test was carried out in 6 cycles, resulting in the nanoemulsion appearance at cycle 0 (a), 1st cycle (b), 2nd cycle (c), 3rd cycle (d), 4th cycle (e), 5th cycle (f); 6th cycle (g).

The freeze-thaw test result showed no significant difference in organoleptics (Fig 2). In addition, droplet size, polydispersity index, pH, viscosity, and curcumin content did not change significantly after six cycles (Table 5). In this test, the dosage form was stored at freeze temperature (-20±2°C) and room temperature (25±2°C).

**Table 5:** pH, droplet size, polydispersity index, viscosity, and curcumin content before and after the freeze-thaw test.

Characteristic	Cycle	
	0	6
Droplet size (nm)	144.62±3.78	148.87±7.02
Polydispersity index	0.30±0.03	0.30±0.08
pH	5.49±0.09	5.36±0.04
Viscosity (cP)	744.20±30.27	788.97±41.12
Curcumin content (%)	98.36±2.51	97.83±3.45

**Fig 3:**
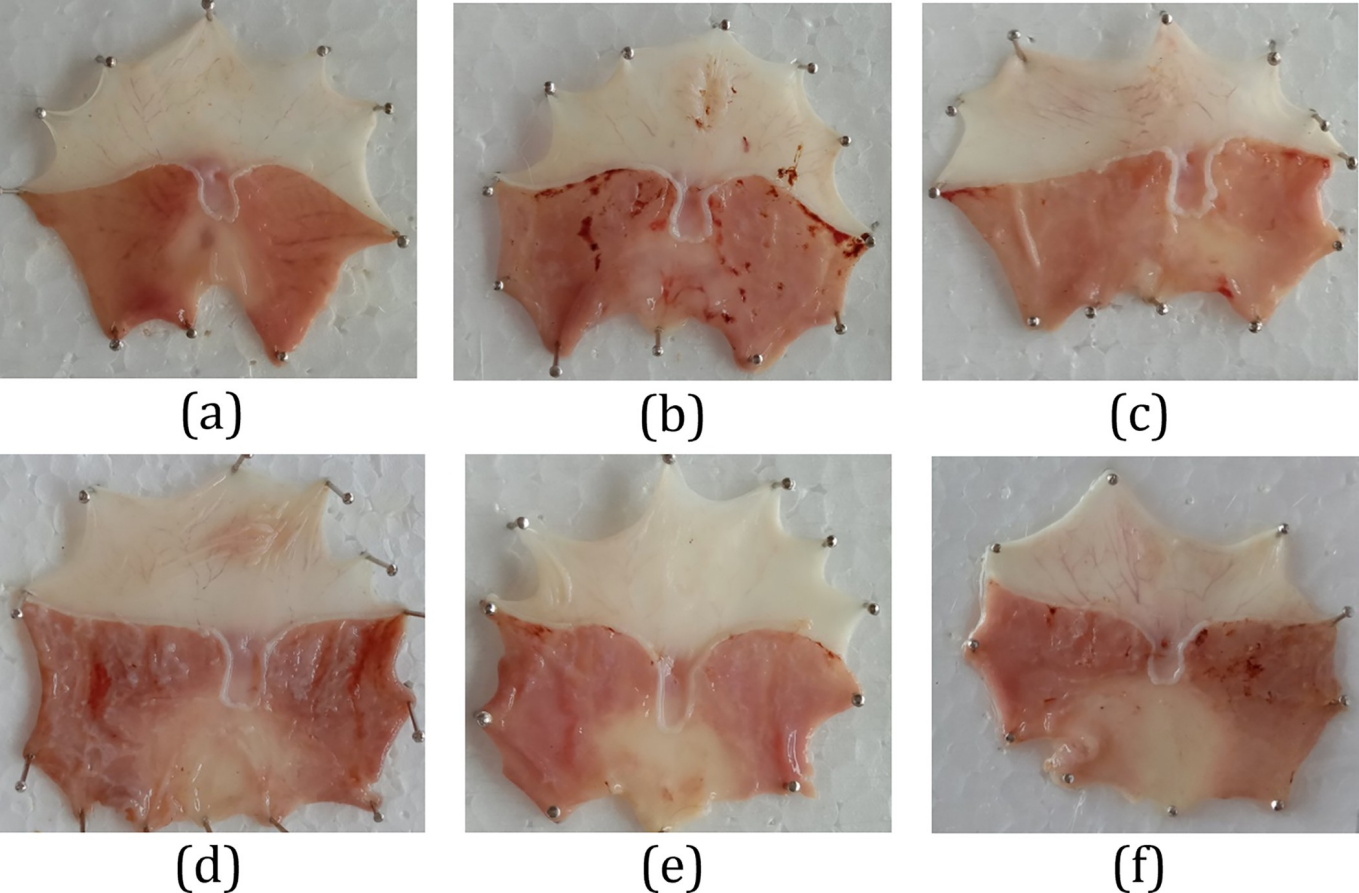
Gastric ulcer macroscopic observation of 6 groups of rats. Macroscopic view of gastric mucosa in healthy control (a); ulcerated control (b); omeprazole 20 mg/kg BW (c); turmeric-black pepper-honey nanoemulsion 1 mL/kg BW (NTBH1) (d); turmeric-black pepper-honey nanoemulsion 2 mL/kg BW (NTBH2) (e); turmeric-honey nanoemulsion (NTH) 2 mL/kg BB (f) groups.

In this study, the activity of nanoemulsion on gastric ulcers was tested in vivo using ethanol-induced gastric ulcers in rats. The rats were fasted for 12 hours before first-day induction. After being treated for six days, the result showed a significant reduction of ulcers in rats treated with turmeric-black pepper-honey nanoemulsion. The results from macroscopic observation showed no hints of ulcer on the gastric mucosal surface of the healthy control group (Fig 3). The ulcerated control group had lesions and ulcer spots along the gastric mucosal surface. On a low dose of turmeric-black pepper-honey nanoemulsion (NTBH1-treated group), many lesions and ulcer spots were still observed along the gastric mucosal surface but with less area than the ulcerated control group. On high-dose turmeric-black pepper-honey nanoemulsion (NTBH2-treated group), the lesions and ulcer spots were much less than in the ulcerated control group. In addition, the lesion and ulcer spots of rats treated with NTH (turmeric-honey nanoemulsion without black pepper) showed fewer lesions and ulcer spots. Still, they had more extensive ulcer areas than the NTBH2-treated group. The sum of the ulcer area and total stomach area was determined using ImageJ. The results from image analysis showed that rats treated with NTBH2 had the lowest ulcer index and score (Table 6). Thus, the curative index of this group is the highest among all groups.

**Table 6:** Ulcer index, curative index, ulcer score, and gastric pH of tested animals.

Group	Ulcer Index	Curative Index	Ulcer Score	pH
Healthy control	0,00	-	0,00	2.80±0.45
Ulcerated control	5.73±3.65	-	4.00±1.22	3.80±1.64
Omeprazole (20 mg/kg of body weight)	1.24±0.56^b^	78.40%	2.10±1.02^b^	4.60±0.89^a^
NTBH1 (1 mL/kg of body weight)	2.17±1.79^b^	62.10%	2.60±0.89	3.40±0.55
NTBH2 (2 mL/kg of body weight)	0.56±0.31^b^	90.15%	2.20±0.45^b^	3.20±0.45
NTH (2 mL/kg of body weight)	1.80±1.47^b^	68.48%	2.40±0.55	3.20±0.45

a = Significantly different compared to healthy control (p<0.05)

b = Significantly different compared to ulcerated control (p<0.05)

NTBH1 = Nanoemulsion containing turmeric, pepper, and honey dose 1

NTBH2 = Nanoemulsion containing turmeric, pepper, and honey dose 2

NTH = Nanoemulsion containing turmeric and honey without pepper

However, the pH of the NTBH1- and NTBH2-treated groups was not significantly different compared to the control group. The results of this study showed that the curative index from the highest to the lowest, i.e., NTBH2, omeprazole, NTH, and NTBH1 (Table 6). The curative index of all treated groups was significantly higher compared to the ulcerated control group. However, the omeprazole- and NTBH2-treated groups showed significantly lower ulcer scores than the ulcerated control group. The result showed no statistically significant difference in gastric pH between all groups except the omeprazole-treated group (Table 6).

**Fig 4:**
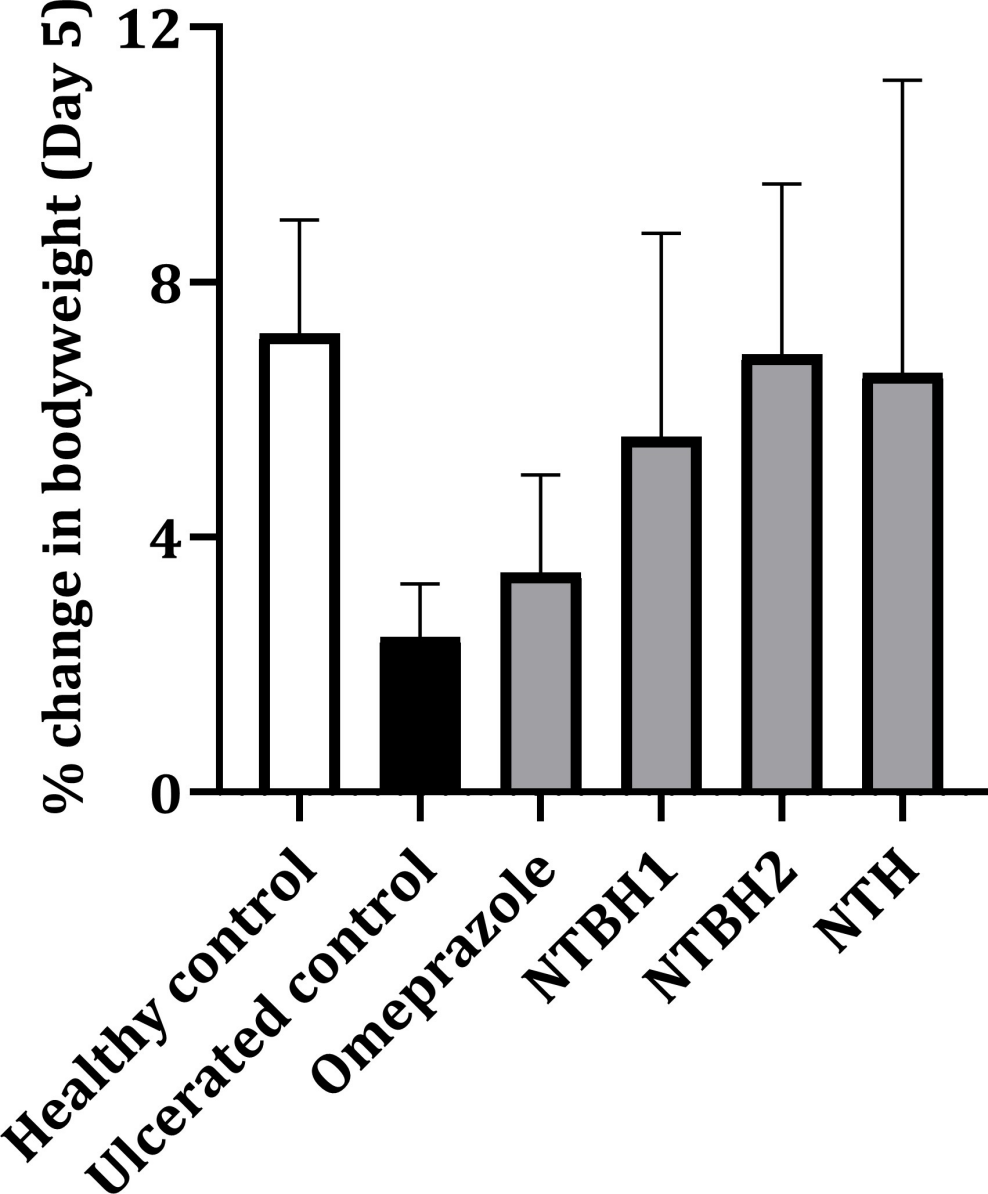
The percentage of body weight gained five days after ethanol induction. The body weight of rats five days after ethanol induction in healthy control, ulcerated control, and groups treated with omeprazole, turmeric-black pepper-honey nanoemulsion 1 mL/kg BW (NTBH1), turmeric-black pepper-honey nanoemulsion 2 mL/kg BW (NTBH2), and turmeric-honey nanoemulsion 2 mL/kg BW (NTH) groups. The percentage was based on the ratio of change in body weight to the initial weight of rats.

The result showed the percentage of body weight change on day five after induction (Fig 4). Each group had no significant difference in initial body weight before day 6 of induction. Thus, the baseline was similar in all groups. On day five after induction, all groups showed increased body weight, but the trend showed that the ulcerated control and omeprazole group had the slightest change compared to other groups. Nonetheless, the difference was not statistically significant.

**Fig 5:**
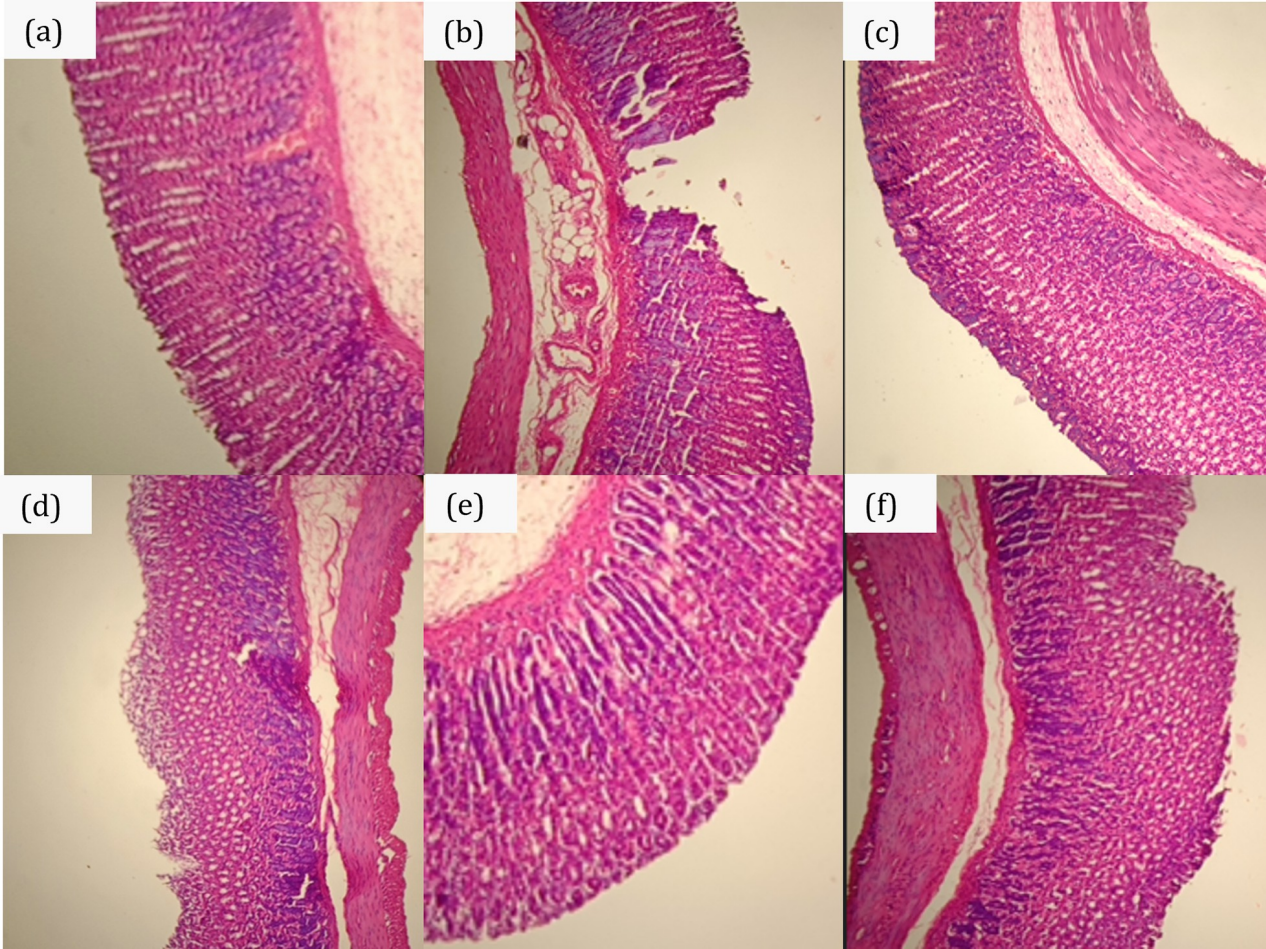
Representative photomicrographs of H&E-stained gastric. Gastric histology of healthy control (a); ulcerated control (b); omeprazole 20 mg/kg BW (c); turmeric-black pepper-honey nanoemulsion 1 mL/kg BW (NTBH1) (d); turmeric-black pepper-honey nanoemulsion 2 mL/kg BW (NTBH2) (e); turmeric-honey nanoemulsion (NTH) 2 mL/kg BB (f) groups.

The histological study of the ethanol-induced gastric ulcer in rats showed severe disruption of the surface epithelium, and necrotic lesions penetrate deeply into mucosa (Fig 5A). The gastric mucosa of the ulcerated control group exhibited significant damage, characterized by severe epithelial erosion and necrosis. The submucosa showed signs of edema, and there were prominent blood vessel dilations, reflecting increased vascular permeability and compromised tissue integrity. The omeprazole-treated group showed mild disruption of surface epithelium mucosa (Fig 5C). The NTBH1-treated group showed lesions in the mucosa, but the surface epithelium has already shown recovery progress (Fig 5D).

Nonetheless, the NTBH2-treated group showed no disruption to the surface epithelium (Fig 5E). In addition, edema and dilated blood vessels were not observed. The NTH-treated group showed a similar histological image as NTBH1, which was in line with the result from gastric macroscopic evaluation.

## Discussion

Adnyana et al. (2014) evaluated the ulcer-curative activity of honey and turmeric on male Wistar rats. The results showed that honey at a lower dosage has a better curative effect than honey at a higher dosage and a combination of honey-turmeric [9]. Honey with a higher dosage was suspected to yield a lesser curative effect due to the acidity of honey, which might counteract its curative properties. A combination of honey-turmeric also showed curative effects, better than turmeric alone but still lesser than honey with a lower dosage [9]. Thus, it was concluded that turmeric has a better effect when combined with honey.

Previous research by Adnyana et al. (2014) administered turmeric infuse orally, and a combination of honey-turmeric was prepared by dispersing honey in turmeric rhizome infuse. Curcumin is the main bioactive compound found in the rhizome of turmeric (*Curcuma longa* Linn.) and in others *Curcuma* spp. [18]. Curcumin is known to have a low bioavailability when consumed orally and does not disperse very well in the water [19, 42, 50]. In this study, honey and turmeric were formulated into liquid dosage forms developed to enhance the bioavailability of curcumin. Curcumin has a relatively high solubility in oil and some organic solvents due to its lipophilicity [52]. Thus, choosing the right oil is essential in this study to enhance the bioavailability of curcumin. In this study, two types of oil were compared to determine which provided the best curcumin solubility, thereby ensuring the highest curcumin loading efficiency in nanoemulsion. Notably, other studies have demonstrated that curcumin exhibits higher solubility in medium-chain triglycerides (MCT) oil [42, 53]. This study utilized a specific type of MCT oil, namely FCO, to assess its effectiveness in curcumin solubility. The results of this study revealed that curcumin exhibited higher solubility in FCO compared to VCO. FCO predominantly contains caprylic acid (C8) and capric acid (C10) triglycerides, whereas VCO is primarily composed of lauric acid (C12) and other long-chain triglycerides, which may hinder its curcumin solubilization capacity [54]. This finding aligns with another study showing that curcumin can be effectively extracted from *Curcuma longa* Linn using MCT oils [55], further supporting the potential of FCO as an effective carrier in curcumin-based nanoemulsion.

In pharmaceutical dosage form, oil is commonly formulated as an emulsion to increase patients’ acceptance of the taste. Nonetheless, emulsions tend to have a white/opaque appearance, making them less attractive for some patients, including children. Nano-sized emulsions have become one of the trending topics and have been extensively studied in pharmaceutical liquid dosage forms. Nanoemulsion is categorized as a colloidal dispersion system with submicron droplet size ranging from 20 to 200 nm [25]. Studies have shown that nanoemulsion can enhance the gastrointestinal absorption of orally administered compounds with low bioavailability [26, 27, 56]. Nanoemulsion may enhance gastrointestinal absorption through various mechanisms, including solubility enhancement, permeability improvement, and increased interaction with the intestinal membrane [26].

In this study, black pepper extract containing piperine was added to the formula to increase the bioavailability of curcumin. The ratio of curcumin and piperine was set to 33:1 in the formula, which was lower than the ratio used by Shoba et al., i.e., 100:1 [21]. This ratio was chosen in our study to ensure that enough piperine is present to maximize the bioavailability of curcumin while maintaining a favorable therapeutic ratio. Studies have shown increased curcumin bioavailability when co-administered with piperine [21, 57, 58]. Piperine is known to inhibit hepatic and intestinal glucuronidation activity [21], and hepatic glucuronidation activity is suspected to be responsible for the low concentration of curcumin in the blood. Thus, inhibiting glucuronidation by piperine might enhance the bioavailability of curcumin [21]. The amount of honey used was also minimized because, according to previous research, the higher dosage of honey might not yield better antiulcer effects [9].

In this study, nanoemulsion was produced using a low-energy method. This is because the nanoemulsion formulated in this study aimed to be made at small and medium industrial facilities that do not have access to sophisticated instruments. FCO was used as an oil phase to produce nanoemulsions due to its higher solubilization capacity for curcumin. FCO contains mostly MCT, which can better dissolve curcumin and create the smallest nanoemulsion droplets compared to other oil types [36, 42]. In addition, nanoemulsions produced with MCT also showed better stability compared to long-chain triglycerides [39]. In MCT-contained nanoemulsion, the smallest droplets could be obtained using Tween 40, Tween 80, and an equal mixture of Tween 20, 80, and 85 as surfactants [59]. The surfactant was first dissolved in the organic/oil phase using the EPI method. Studies have shown that the initial surfactant location (aqueous vs. organic phase) can influence the droplet size of the nanoemulsion produced with the EPI method [36]. In this study, Tween 80 was used as a surfactant with various surfactant-to-oil ratios (SOR), i.e., 1 (Formula F1G1), 1.5 (Formula F2G1A) and 2 (Formula F3G2A, F3G2C). Tween 80 is a non-ionic surfactant used mainly in nanoemulsion formulation using a low-energy method [36]. The results showed that the smallest droplet size was obtained from formula F3G2A, which contains glycerin as cosolvent, twice the amount of Tween 80. Surfactant concentration influences the value of the critical water concentration where phase inversion occurs and the size of the oil droplets produced [36]. Thus, the result in this study was in line with the result observed in another study where smaller inner oil droplets in multiple emulsions (O/W/O) could be formed during the titration process at relatively higher surfactant concentrations [36].

Glycerin was used as a cosolvent in the nanoemulsion formula because it has the ability to modify the physicochemical properties of the aqueous solutions, including viscosity, density, and interfacial tension [60]. The addition of glycerin has been used to reduce the amount of surfactant required to form microemulsion and increase the storage stability of oil-in-water emulsion [60, 61]. In this study, the increased concentration of glycerin in the formula decreased the droplet size from 341.2±20.3 nm in formula F3G1A to 144.6±3.8 nm in formula F3G2A2. Both formulas used a similar amount of Tween 80, but the latter used twice the amount of glycerin. In addition, both formulas used similar amounts of sodium alginate as a viscosity enhancer.

Two types of gelling agents, sodium alginate and sodium carboxymethylcellulose, were evaluated and compared for their effectiveness in increasing the viscosity of nanoemulsion while preserving the nanoscale droplet size. Both natural gelling agents are polysaccharides commonly used to enhance the viscosity of the oral liquid dosage form. A viscosity enhancer was added to improve the nanoemulsion storage stability because it can create steric and electrostatic repulsion between droplet interfaces [43]. Thus, droplet aggregation in the nanoemulsion system can be prevented. In addition to stability improvement, adding a viscosity enhancer also influenced the droplet size. The result showed that CMC sodium-added nanoemulsion (formula F3G2C) had a bigger droplet size than the formulas that used sodium alginate as a viscosity enhancer (formula F3G2A). Therefore, sodium alginate was further used in this study as a viscosity enhancer for nanoemulsion system.

A stability test was carried out to ensure that the nanoemulsion system can keep the droplet size at the nanosize range and, most importantly, to ensure the stability of curcumin. There were two types of stability tests, i.e., storage test at 25°C and 40°C and freeze-thaw test. Nanoemulsion containing honey, turmeric, and black pepper was observed for four weeks of storage. A droplet size change was observed at both temperatures, but it still fulfilled the criteria for nanoemulsion [51]. Nonetheless, the polydispersity index did not change significantly during storage at both temperatures. The polydispersity index indicates the homogeneity of nanoemulsion droplet size, and the homogenous system has a polydispersity index close to zero [62]. During storage at 40°C, the nanoemulsion viscosity decreased significantly. The reduced viscosity might cause nanoemulsion to become unstable due to excessive movement of particles and cause nanoemulsion droplets to join. The reduced viscosity might also explain the increased droplet size when stored at 40°C compared to 25°C. The pH of the nanoemulsion also slightly decreased, but still in the range of pH stability for curcumin [63].

A freeze-thaw test was carried out to evaluate the stability of nanoemulsion by subjecting the sample to six cycles of freezing and thawing. The droplet size, PDI, pH, and curcumin content did not change significantly after six cycles, indicating the physical and chemical stability of the formulation. The nanoemulsion viscosity slightly increased, but the change was not statistically significant, suggesting that the nanoemulsion maintained its rheological properties under stress conditions. These findings align with other studies showing the nanoemulsion potential for stability during storage and handling [64, 65].

An ethanol-induced ulcer model was used to evaluate the antiulcer activity of nanoemulsion. Ethanol, administered orally, can penetrate gastric mucosa by solubilizing gastric protective mucosa, causing gastric mucosa to be exposed and vulnerable to proteolytic and hydrolytic activities [17]. Ethanol also causes lesions on gastric mucosa, lowering the secretion of bicarbonate and protective mucous [17]. Acute inflammatory reactions will also occur around the damaged mucosa [66]. Ethanol also decreases blood circulation around the stomach and induces oxidative stress due to the formation of reactive oxygen species [67]. In this study, ethanol was administered for six days to mimic the chronic gastric injury. This model was adapted from Hernández-Muñoz et al. with few modifications [16, 17]. Repeated exposure to ethanol over a subchronic period caused histological evidence of gastritis and modifications at the plasma membrane level [16, 17]. Thus, this model is suitable for evaluating the anti-ulcer activity in pre-established gastric ulcers.

Macroscopic observation of gastric mucosa showed that the mucosa of the healthy control group remained intact and healthy. In contrast, the administration of 96% ethanol to induce ulcers caused inflammation and gastric mucosal damage, as seen in the ulcerated control group. The inflamed gastric tissue exhibited typical features of inflammation, including reddened mucosa due to increased blood flow as part of the inflammatory response, as well as lesions where both the mucosa and submucosa are damaged. These observations align with findings from other studies using [16, 17]. Treatment with omeprazole, NTBH1, NTBH2, and NTH in ethanol-induced ulcerated rats reduced excessive mucosal damage and helped maintain the integrity of the gastric mucosa.

Analysis of the ulcer index and score parameter was done with a one-way ANOVA method (p<0.05) using GraphPad Prism 9. The analysis result showed that the obtained data passed the normality test, and there is a significant difference between the ulcerated control group and all treated groups, i.e., omeprazole-, NTBH1, NTBH2, and NTH-treated groups. The lowest ulcer index and score were found in NTBH2-treated groups, even lower compared to a positive control group treated with omeprazole. The NTBH2 group was treated with turmeric, black pepper, and honey nanoemulsion with twice the amount of NTBH1. The NTBH1-treated group also showed decreased ulcer index and score, but the ulcer parameters were still higher than the omeprazole-treated group. This dose-dependent improvement suggests that the higher concentration of active compounds, i.e., turmeric, black pepper, and honey, may enhance the therapeutic potential of nanoemulsion.

Although all treated groups showed healing effects on ulcerated rats, the omeprazole-treated group showed a higher pH of the gastric liquid. The gastric pH was significantly higher compared to the healthy control group. This result is in accordance with the known mechanism of omeprazole as a proton pump inhibitor (PPI). It decreases the amount of acid the stomach produces, thus increasing the intragastric pH [4]. The increased gastric pH and the long-term use of PPI have been associated with side effects, such as vitamin and mineral deficiencies, enteric, respiratory, and urinary tract infections, hypochlorhydria that may induce hypergastrinemia, etc. [68].

The increased gastric pH was not observed in all nanoemulsion-treated groups. This is because turmeric and honey have different mechanisms in reducing gastric ulcers. As discussed in our previous study, the ability of honey to reduce ulcers can be explained by its antioxidant and anti-inflammatory activities that are contributed by flavonoids and phenolic compounds [9, 69]. Curcumin in turmeric also contributes to antioxidant and anti-inflammatory activities. These activities can reduce ulcers by stimulating epithelization and remodeling effects and regulating matrix metalloproteinase [9, 70]. Nonetheless, these activities of curcumin will never be followed if it is not absorbed into the systemic circulation. Therefore, the nanoemulsion system in this study is critical in delivering curcumin to enhance its bioavailability and ulcer healing activity.

The reepithelization activity of curcumin-containing nanoemulsion can be observed in the photomicrographs of H&E-stained gastric tissues. The histological features observed in the ulcerated control groups were consistent with the pathological effects of ethanol, which is known to induce gastric mucosal injury through the generation of reactive oxygen species and the disruption of mucosal protection mechanisms [13, 14]. The epithelial layer displayed moderate reepithelization in the omeprazole-treated group, with reduced ulceration compared to the ulcerated control group. The results suggest that omeprazole exerted its protective effect through acid suppression, consistent with its known mechanism of action [15]. The NTBH2-treated group showed the most advanced healing, with a nearly restored mucosal layer, minimal inflammatory infiltration, and a well-formed gastric architecture. This is likely due to the combined antioxidant and anti-inflammatory effects of curcumin, the bioenhancing properties of piperine, and the reparative actions of honey, which promote cellular regeneration and reduce oxidative stress [18, 20, 62]. Histological findings corroborated the macroscopic observation, supporting the conclusion that NTBH2 offers superior healing of ethanol-induced gastric ulcers.

In this study, we also evaluated the effect of black pepper on nanoemulsion antiulcer activity. We included the NTH group, which was treated with turmeric and honey nanoemulsion without adding black pepper. The honey and turmeric in the NTH group were in similar amounts as in the NTBH2 group. Yet, the NTH showed higher ulcer parameters than the NTBH2 group, indicating the role of black pepper extract in reducing the ulcer in ethanol-induced gastric ulcer rats. According to macroscopic observations and histological findings, the NTH group showed curative effects like those of the NTBH1 group. Thus, it can be concluded that black pepper enhanced turmeric’s pharmacodynamic activities, which might be explained by its ability to increase the bioavailability of curcumin. Piperine, the main component of black pepper, is known as a bio-enhancer that can improve the bioavailability and bioefficacy of the drug by intervening in its metabolism [71]. Furthermore, piperine is also known to have gastroprotective effects by inhibiting oxidative processes [22], thereby contributing to its anti-ulcer activity in the NTBH2-treated group.

The administration of nanoemulsion appeared to increase weight gain better than that of the standard control and ulcerated control groups. Carbohydrate contents in honey from the nanoemulsion appeared to contribute as an additional energy source for the rats. The nanoemulsion also seemed to possess excellent anti-ulcer activity and, hence, might be able to prevent weight loss, which often happens to individuals with gastric ulcers [72].

To conclude, the combination of turmeric, black pepper, and honey formulated into nanoemulsion using FCO as oil phase, Tween 80 as surfactant, and glycerin as cosolvent showed stability during storage and healing activity on the ethanol-induced gastric ulcer in rats. The results of the efficacy evaluation of nanoemulsion highlight the critical role of dose optimization in maximizing the therapeutic benefits of nanoemulsion formulations. In addition, the results underscore the potential of combining natural products in a synergistic manner to improve efficacy. The observed anti-ulcer effects of NTBH2 nanoemulsion result from a complex interplay of molecular and cellular mechanisms. The nanoemulsion formulation enhances the bioavailability of curcumin, enabling it to exert its antioxidant and anti-inflammatory effects more effectively. Piperine enhances the bioavailability of curcumin and contributes its gastroprotective effects, while honey promotes mucosal healing and tissue regeneration through anti-inflammatory and growth-stimulating properties. Together, these components act in concert to reduce gastric mucosal damage, promote healing, and restore the integrity of the gastric epithelium. Nanoemulsion containing turmeric, black pepper, and honey is considered safe because it did not affect the body weight of the rats. Hence, it can be used for long-term therapy for gastric ulcers. However, sub-chronic and chronic toxicity studies need to be carried out to ensure the safety of this dosage form for long-term use.

## Supporting information

S1 File (ZIP)
